# Optical Coherence Tomography in a Cohort of Genetically Defined Hereditary Spastic Paraplegia: A Brief Research Report

**DOI:** 10.3389/fneur.2019.01193

**Published:** 2019-11-22

**Authors:** Marinela Vavla, Gabriella Paparella, Alessandro Papayannis, Riccardo Pascuzzo, Giulia Girardi, Francesco Pellegrini, Gianluca Capello, Gianni Prosdocimo, Andrea Martinuzzi

**Affiliations:** ^1^SOS Neuromotor Unit, Scientific Institute, IRCCS E. Medea, Conegliano, Italy; ^2^Department of Ophthalmology, AULSS2 Marca Trevigiana, Treviso, Italy; ^3^Neuroradiology Unit, Fondazione IRCCS Istituto Neurologico Carlo Besta, Milan, Italy

**Keywords:** hereditary spastic paraplegia, optical coherence tomography, biomarker, retinal nerve fiber layer, longitudinal

## Abstract

**Introduction:** *In-vivo* objective documentation of pathological changes in neurodegenerative disease is a major aim to possibly improve our ability to monitor disease progression and response to treatment. Temporal thinning of the retinal nerve fiber layer (RNFL) thickness shown by spectral domain optical coherence tomography (SD-OCT) has been reported in association with the complex forms in hereditary spastic paraplegia (HSP). We performed an assessment of the RNFL thickness in a group of HSP patients, including a longitudinal follow-up in a subgroup. Our aim was to measure and compare the changes and correlate them to clinical progression.

**Materials and Methods:** Twenty-three HSP patients were recruited and studied with the SD-OCT including papillary and macular scan by Spectralis. The clinical severity was assessed using the Spastic Paraplegia Rating Scale.

**Results:** Thinning of the superior, nasal and inferior quadrants bilaterally were observed compared to the normative data in both pure and complicated forms, that were clearly pathological only in a proportion of cases. Thinning correlated with age and disease duration, but not with clinical severity. The longitudinal study (*n* = 9) showed no significant change compared to the baseline data for the period of observation (mean 10.7 months).

**Conclusions:** RFNL is frequently thinned in HSP with no specific recognizable pattern of quadrants involved and SPG types. The small sample size and the short follow-up time showed no clear progression. Although SD-OCT appraisal of RFNL deserves to be explored in neurodegenerative conditions, it might not be suitable for use as a biomarker in HSP as it appears not to be specific to this condition and can be a feature of aging.

## Introduction

Nowadays, there is unstoppable exponential research for therapeutic opportunities in a wide range of neurological diseases. An urgent need for sensible biomarkers emerges as further improvements in the diagnostic processes of neurodegenerative conditions are being made. A biomarker is an objectively measured characteristic that reflects any normal or pathological process or pharmacological response in the living organism (1). The biomarkers are theoretically intended to reflect diagnostic accuracy, provide insight in the early diagnosis and follow-up, be useful in clinical trials as outcome measures, be reasonably standardized and easily interpreted (2). In human diseases and particularly in neurodegenerative conditions, biomarkers could fill several gaps and provide assistance with the diagnostic process, allowing pre-symptomatic diagnosis and resolution of cases with atypical presentation (3). Finally, efficient biomarkers could precisely measure changes induced by treatment in a time-frame compatible with the usual clinical trials duration (6–12 months), improving and speeding up treatment discovery (4).

The hereditary spastic paraplegia (HSP) is a progressive neurodegenerative condition presenting with lower limb spasticity, weakness and axonopathy of the long corticospinal tracts (5). It is classified in pure and complex forms (6), with the latter forms showing additional neurological (cerebellar symptoms, neuropathy, cognitive impairment, amyotrophy, parkinsonism, and leukodystrophy) and non-neurological signs (cataract, ichthyosis, and short stature) (7). HSP is part of a large and molecularly heterogeneous group of diseases with autosomal dominant, recessive, x-linked and maternal inheritance, counting over 80 genetic loci and over 60 identified causative genes (8). There is still no treatment available for this condition.

The extreme genetic heterogeneity, the variability in age of onset (from early infancy to late adulthood), disease progression and ancillary clinical signs, make HSP a condition for which biomarkers could be extremely useful, especially if non-invasive, convenient, easily accessible, replicable in common settings, expressed in precise metrics, and possibly capable to specifically label the condition (9). HSP has been studied at many levels, from the cellular up to the organs and systems level with a progressive understanding of the condition. The various studies are depicting a picture of complex and converging mechanisms that involve many systems, well-beyond the main target of the corticospinal tracts. We have already shown that by systematic application of neurophysiology and advanced MRI techniques, significant changes can be detected in HSP patients in measures of integrity of both motor and sensory long traits (10). The optic pathway is a highly specialized part of the central nervous system (CNS). The anatomical and functional integrity of the nerve fibers can be assessed at various levels from the retina to the cortex. The retinal nerve fiber layer (RNFL) thickness has been studied by spectral-domain optical coherence tomography (SD-OCT), an easy and cheap tool implemented in the everyday clinical practice. The RNFL thickness may be exploited as a useful clinical measure in HSP and could also provide an easily accessible window to the optic nerve pathology.

Only two reports have provided insights on the RNFL thickness in HSP patients. Out of a consistent cohort of HSP, RNFL thinning was found only in the complex forms of HSP (SPG7) and, in particular, in the temporal and temporal-inferior sectors (11). These findings, and, in particular, the SPG7 RNFL thickness loss, were confirmed in a subsequent case report with a selective reduction of ganglionic cells, with diffuse and severe reduction of the papillar nervous fibers (12). A more systematic and longitudinal appraisal of RNFL thickness in a larger variety of HSP could help in understanding its potential use as biomarker for HSP, especially if timed according to the typical time-frame of a randomized clinical trial that is usually within 12 months.

Here, we report a prospective cross-sectional assessment of the RNFL thickness in a cohort of genetically heterogeneous HSP patients and a longitudinal study in a subgroup of this cohort. Our aim was to measure the RNFL thickness and to explore a possible association between measured changes and clinical status and progression.

## Methods

### Patients

The patients were recruited at the Scientific Institute “Eugenio Medea” from June 2015 until February 2017. Patients entered the clinic for a complete clinical and diagnostic assessment. HSP diagnosis was based on genetic confirmation of SPG type. In total, 23 patients were recruited and underwent a baseline SD-OCT scan and clinical assessment. The inclusion criteria consisted of genetic diagnosis of HSP, absence of any opthalmological comorbidities, and consent to participate in the study. Nine patients underwent a second SD-OCT scan 6–12 months after the baseline one. The patients were given information regarding the study and gave written informed consent prior to their inclusion in the study. The study protocol was approved by the Ethics Committee of Veneto Regional Institution (# 155/CE, 28/05/2015) and was conducted in accordance to the ethical standards of the Declaration of Helsinki (13).

### Clinical Assessment

Each patient underwent a full neurological examination by an experienced neurologist (AM). The Spastic Paraplegia Rating Scale [SPRS, (14)] was administered by an experienced physiotherapist (GG). Age at onset (AAO) was considered as the age when first HSP-related signs and symptoms occurred. Disease duration was considered as the time elapsed between the AAO and the age at the SD-OCT scan.

### Retinal Imaging by Spectral-Domain Optical Coherence Tomography

The subjects in this study underwent a SD-OCT examination using the Heidelberg Engineering Spectralis SD-OCT (Heidelberg Engineering, Heidelberg, Germany, Spectralis EYE Explorer version 1.10.20). This SD-OCT utilizes a scanning superluminescence diode to emit a scan beam with a wavelength of 870 nm to acquire 40,000 A scans/s with a depth resolution of 7 μm, from which various retinal layers can be identified, and objectively and precisely assessed. The SD-OCT device also combines SD-OCT and confocal infrared laser ophthalmoscope, which provides a reference infrared fundus image. The SD-OCT parameters calculated for this study were mean RNFL thickness around the optic nerve in nasal (NAS), inferior (INF), temporal (INF), and superior (SUP) quadrant of both eyes. The mean retinal thickness was measured in μm per quadrant of the peripapillary area. All scans were performed by experienced ophthalmologists (AP, FP, and GC). The images were subsequently reviewed for acceptable signal strength, correct placement of the scan ring, and appropriate beam placement.

### Statistical Analysis

Statistical analysis was undertaken using IBM SPSS Statistics for Windows, version 23. Demographic, clinical and RNFL thickness data were presented as mean, standard deviations and range of minimum and maximum. The RNFL thickness values of each quadrant were transformed into Z-scores, based on the corresponding mean and standard deviation of the normative values from the healthy subjects of a previous report (15), that were used as control group of this study. We classified the Z-scores as borderline and pathological if lower than −2 and −3, respectively, otherwise they were considered normal. Comparison of groups and number of impaired (i.e., borderline or pathological) values of RNFL thickness in all the quadrants bilaterally was presented as frequency (%). Binomial two-tailed test was used for sex and HSP form (pure vs. complex) ratios. Fisher's test was adopted to analyze any differences in the proportion of borderline and pathological patients between the genetic groups and between HSP pure and complex forms. Spearman's coefficient was used to study the correlation between the clinical and the eight RNFL thickness variables, controlling the false discovery rate (FDR) with Benjamini-Hochberg procedure. Two tails *t*-test was performed to compare all the RNFL thickness of the four quadrants bilaterally at baseline between patients and normative values, controlling the FDR with Benjamini-Hochberg procedure. The *t*-test gaussianity assumption for each of the RNFL thickness variables of the patient group was checked and confirmed (all *p* > 0.05) by the Shapiro-Wilk test. Longitudinal data were compared with a non-parametric test (Wilcoxon signed-rank test) due to limited sample size.

## Results

### Descriptive Statistics

Twenty-three patients recruited underwent SD-OCT scans on both eyes. The clinical, demographic, and RNFL thickness data are presented in Table 1. All the patients had confirmed genetic diagnosis of HSP, in particular SPG4 (*n* = 8), SPG3a (*n* = 6), SPG72 (*n* = 3), SPG5 (*n* = 2), SPG7 (*n* = 2), and SPG8 (*n* = 2). All the patients were free of any comorbid opthalmological conditions such as cataract, glaucoma and maculopathy. Mean age at the SD-OCT scan was 44.63 years with a range of 14.42–76 years old. Disease severity ranged from mild to moderate with SPRS values from 3 to 38. There was a higher frequency of female patients (56.5%) vs. males and of pure forms (60.9%) when compared to complex ones. However, the proportion of females did not differ significantly from the proportion of males (binomial two-tailed test, *p* = 0.678). Similarly, no difference was observed between the pure and complex forms (binomial two-tailed test, *p* = 0.405).

**Table 1:** Demographic, clinical, and RNFL thickness data of the cohort.

**Parameters**	**Mean ± SD**	**Min–max**
Sex	13F:10M	
AAO (y)	26.56 ± 21.84	0–74
DD (y)	18.06 ± 12.55	2–50
AA sd-oct (y)	44.63 ± 17.7	14.42–76
SPRS (0-52)	24.3 ± 9.39	3–38
P:C	14P:9C	
Genotype (*n*)	SPG3A (6) SPG4 (8) SPG5 (2) SPG7 (2) SPG8 (2) SPG72 (3)	

*F, female; M, male; SD, standard deviation; AAO, age at onset; DD, disease duration; AA SD-OCT, age at SD-OCT scan; P, pure; C, complex; SPRS, Spastic Paraplegia Rating Scale; RNFL, Retinal Nerve Fiber Layer; R-SUP, right superior; R-INF, right inferior; R-TEMP, right temporal; R-NAS, right nasal; L-SUP, left superior; L-INF, left inferior; L-TEMP, left temporal; L-NAS, left nasal; y, years. Normal values refer to Naithani et al. (15)*.

The Supplementary Table 1 reports the RNFL thickness raw data pointing out the normal values (green) and impaired values registered as borderline Z score < −2 SD (yellow) and pathological Z score < −3 SD (red) in all the quadrants bilaterally in the study cohort (*n* = 23). About 60.86% of the patients (*n* = 14) showed no impairments in any of the quadrants bilaterally. The remaining cohort showed impaired values in 2 quadrants (17.39%, *n* = 4), in 1 quadrant (13.04%, *n* = 3), in 4 quadrants (4.34%, *n* = 1), and in 5 quadrants (4.34%, *n* = 1). Overall, by considering the number of quadrants in the cohort (*n* = 184 quadrants), only 20 (10.86%) quadrants values fell into the pathological and borderline ranges. The pathological values were measured at the following quadrants: *n* = 3 in right-superior (R-SUP), *n* = 1 in the left-superior (L-SUP) and *n* = 1 in left-inferior (L-INF). The borderline values measured were reported at the following quadrants: *n* = 4 in L-SUP, *n* = 4 in L-INF, *n* = 3 in R-SUP, *n* = 3 in R-INF, and *n* = 1 in L-NAS.

### Subgroup Comparisons

The impairments were present in both pure and complex forms, with no significant differences between the two groups in the proportion of patients with impairments in any quadrant (Fisher's test, all *p* > 0.05).

**Figure 1:**
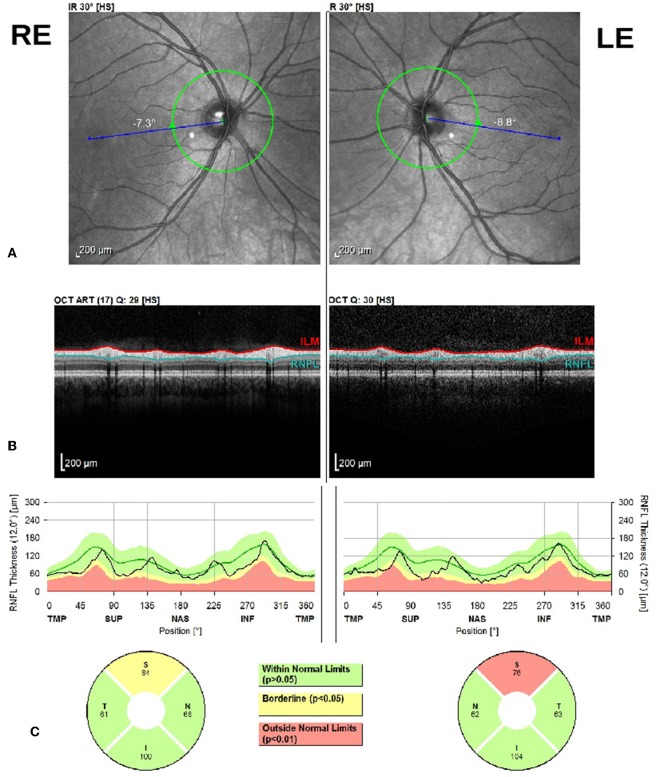
Bilateral optic disc RNFL scan with Heidelberg Spectralis SD-OCT. From the top to the bottom: **(A)** Right Eye (RE) and Left Eye (LE) eye fundus infrared image (IR), **(B)** peripapillary SD-OCT scan, **(C)** RNFL thickness report by quadrant for both eyes.

Within the pure forms, the SPG4 subgroup (*n* = 8) showed impairments in three cases (37.5%) in R-SUP, two (25%) in L-SUP, and one (12.5%) in L-INF (Figure 1). The SPG3a subgroup (*n* = 6) showed impairment in only one patient in 5 quadrants: R-SUP, L-SUP, R-INF, L-INF, and L-NAS. Fisher's test showed no significant differences between the SPG3a and SPG4 proportions of impairments in any quadrants (all *p* > 0.05).

The size of the remaining subgroups was too limited to perform any statistical test. However, SPG72 (*n* = 3) showed pathological value in one patient at R-SUP quadrant. The SPG7 subgroup (*n* = 2) showed borderline values in one patient at the SUP and INF quadrants bilaterally. The SPG5 subgroup (*n* = 2) showed pathological and borderline bilateral INF quadrants in only one patient. The SPG8 subgroup (*n* = 2) showed only borderline values in the L-SUP and L-INF in only one patient.

### RNFL Thickness Variation From Controls

The results of the comparison between the RNFL thickness and the normative is shown in Table 2. We observed a statistically significant reduction of the SUP (*p* < 0.0001 both right and left), INF (*p* = 0.0043 right, *p* = 0.0038 left), and NAS (*p* = 0.0022 right, *p* = 0.0007 left) quadrants RNFL thickness with respect to the normative data. No statistically significant variation was observed at the TEMP quadrants (*p* = 0.0525 right, *p* = 0.6743 left). The same conclusions can be drawn after correction for multiple comparisons with FDR method.

**Table 2:** RNFL thickness in different quadrants in the cross-sectional study (*n* = 23).

	**Mean ± *SD***	**Min; max**	***T***	**Dof**	*p*-value^*^	**Adjusted *p*-value with FDR**
N-SUP	126.4 ± 13.78					
R-SUP	108 ± 14.35	80; 126	5.3833	81	**<0.0001**	**<0.0001**
L-SUP	111.96 ± 15.87	76; 134	4.0964	81	**<0.0001**	**0.0004**
N-INF	125.45 ± 13.48					
R-INF	115.43 ± 14.99	86; 143	2.9364	81	**0.0043**	**0.0058**
L-INF	114.65 ± 17.72	78; 143	2.9844	81	**0.0038**	**0.0058**
N-TEMP	64.32 ± 11.1					
R-TEMP	69.70 ± 11.23	45; 94	1.9682	81	0.0525	0.0600
L-TEMP	65.39 ± 8.03	44; 76	0.4218	81	0.6743	0.6743
N-NAS	81.6 ± 20.94					
R-NAS	65.87 ± 18.23	40; 112	3.1692	81	**0.0022**	**0.0043**
L-NAS	64.17 ± 17.61	36; 109	3.5368	81	**0.0007**	**0.0018**

* *Two tails t-test, comparing baseline values to the control group data in Naithani et al. (15). Significant differences (P < 0.05) are in bold. FDR, false discovery rate; RNFL, Retinal Nerve Fiber Layer; R-SUP, right superior; R-INF, right inferior; R-TEMP, right temporal; R-NAS, right nasal; L-SUP, left superior; L-INF, left inferior; L-TEMP, left temporal; L-NAS, left nasal*.

### Correlations

The relationship between demographic, clinical data and RNFL thickness for all the quadrants of both eyes is presented in Supplementary Table 2. Significant correlations were found between disease duration and right INF quadrant RNFL thickness (rho = 0.574, *p* = 0.004) and the left TEMP quadrant RNFL thickness (rho = 0.436, *p* = 0.037). In addition, a significant correlation was observed between the age at the SD-OCT scan and the left INF nasal quadrant RNFL thickness (rho = 0.516, *p* = 0.012). Other parameters such as age at onset and disease severity (SPRS) showed no significant correlation with the RNFL thickness of all the quadrants bilaterally. However, none of the above correlations is significant after correction for multiple comparisons with FDR method.

### Longitudinal Data

A subgroup of 9 patients underwent a follow-up SD-OCT scan on average 10.7 months after the baseline: the follow-up RNFL thickness showed no significant change in any of the quadrants bilaterally with respect to the baseline (Table 3, Supplementary Figure 1).

**Table 3:** RNFL thickness variation in the longitudinal study (*n* = 9).

	**Baseline** **Mean ± SD**	**Follow-up** **Mean ± SD**	***W***	*p*-value^*^
R-SUP	102.44 ± 14.27	102.33 ± 17.01	24.5	0.3997
L-SUP	103.33 ± 17.17	105.22 ± 14.53	30.5	0.3722
R-INF	106.33 ± 14.14	102.33 ± 11.55	16.5	0.5128
L-INF	100 ± 16	101.11 ± 15.10	18	0.5541
R-TEMP	66.22 ± 13.70	66.78 ± 14.33	27	0.6329
L-TEMP	60.11 ± 7.74	61.33 ± 8.47	20	0.3499
R-NAS	60.22 ± 12.25	60.56 ± 10.21	22	0.6224
L-NAS	56.56 ± 11.76	58.22 ± 10.97	32.5	0.2595

* *Wilcoxon signed-rank test, comparing baseline and follow-up data. RNFL, Retinal Nerve Fibre Layer; R-SUP, right superior; R-INF, right inferior; R-TEMP, right temporal; R-NAS, right nasal; L-SUP, left superior; L-INF, left inferior; L-TEMP, left temporal; L-NAS, left nasal*.

## Discussion

In this study we have performed a systematic paraclinical appraisal of various HSP forms in search for efficient biomarkers in the retina. Previous studies have indicated the optic system, and, in particular, the RNFL, as a possible target of the disease process at least in some complicated HSP (notably SPG7) (12). Here we show that the involvement of the optic system, at least in the form of a thinning of the RNFL, is not a constant feature of complex HSP but is more widespread and extended also in the pure forms. The observed thinning is mostly mild to moderate and does not affect any specific quadrants, but notably spares the temporal one.

A small proportion of the HSP cohort reported clearly pathological values, particularly in the left SUP quadrants. Conversely, borderline values were registered in all the SUP and INF quadrants bilaterally. More than half of the patients showed a tendency from borderline to pathological values in at least one or two quadrants. Our cross-sectional data showed a RNFL thinning in the SUP, INF and NAS quadrants bilaterally. Surprisingly, the TEMP quadrants were spared. No correlations were observed between the RNFL thickness and the clinical data, which confirm previous findings (11).

RFNL thinning in the TEMP quadrant is often reported in optic neuropathies, and it is a hallmark of mitochondrial dysfunctions (16). It seems that in HSP the process of retinal ganglion cells damage is different, resulting in preferential involvement of other retinal fiber bundles. The extension of the HSP related pathology to a non-corticospinal system deserves further systematic study in larger cohorts and for a longer follow-up period to better characterize the pattern and to explore the parthenogenesis.

By analyzing the most represented genotype subgroups, we observed impaired values in the nasal quadrants bilaterally in SPG3a and in the SUP quadrants bilaterally in the SPG4. However, no significant differences emerged between the two groups. Interestingly, our two SPG7 patients showed both borderline values in various quadrants bilaterally. These findings, among the genotype differences, should be considered cautiously considering the small sample size. However, the genotype specific effect could eventually weight on the outcome as pointed out elsewhere (11, 12). Perhaps the mutations influence in accelerating the age-related phenomenon of neuronal loss in the retina, as suggested by the trend of association between RNFL results and disease duration.

The RNFL thickness has been proposed as a biomarker useful to monitor the neurodegeneration in many conditions. There have been controversial results in Alzhemier's Disease (AD) regarding the use of RNFL thickness as a biomarker (17, 18). Hypothesis to use this measure for a preclinical screening (19) or any association to cognitive impairment markers in AD (20) have let to inconclusive results. Cross-sectional studies in Parkinson's Disease show an overall thinning of RNFL thickness (21).

So far, there have been few cross-sectional studies in hereditary ataxias investigating the RNFL thickness as a diagnostic and biomarker measure. In particular, studies in Friedreich's ataxia (FRDA) showed correlations of RNFL thickness to clinical data such as age at onset, disease duration, clinical severity, the patients' reported outcome measure, and GAA triplet expansion (22–25). This is the first longitudinal study in HSP we are aware of, and no RNFL thickness variation in the time between the follow-up and baseline assessments. Supposedly, this short follow-up period was inadequate to detect any significant change in damage of this CNS district, but the choice was made to match the typical duration of randomized clinical trial (6–12 months). Similarly, in a recently completed clinical trial in FRDA where the longitudinal measurement of RNFL thickness was performed, there was no differences either during the 6 months treatment period or in follow-up (ClinicalTrials.gov Identifier: NCT03888664; Paper submitted). Interestingly, a 3-year longitudinal prospective study on a large cohort of patients with relapsing-remitting Multiple Sclerosis identified cut-off annual peripapillary RNFL thinning rates, that could work as a fit proposal of a biomarker for neurodegeneration monitoring (26).

The major limitation of our study is the small number of subjects and their genetic heterogeneity. However, the main aim of this exploratory work was to understand the potential usefulness of SD-OCT as a supporting measure to document extra-motor and extra-neural involvement in HSP and its progression, and if there was any specific subgroup of patients to be targeted and within which time-frame. Although the size of the cohort is small, we believe we provide evidence that RNFL thinning may occur in about any HSP form. Further investigations of this outcome measure for a period of time longer that 1 year could highlight the feasibility of SD-OCT as an indicator of disease progression.

In conclusion, our findings show that all the eye quadrants can be affected in HSP with no discrimination for the clinical presentation (pure or complex) of the disease. Considering the retina as an extension of the CNS, it is interesting to understand whether the RNFL impairment is disease specific or simply an extension of the degenerating brain. The time-dependent progression rates should be further investigated by designing prospective longer and more inclusive longitudinal studies.

## Data Availability Statement

The datasets generated for this study are available on request to the corresponding author.

## Ethics Statement

The studies involving human participants were reviewed and approved by Ethics Committee of Veneto Regional Institution. Written informed consent to participate in this study was provided by the participants' legal guardian/next of kin.

## Author Contributions

AM: conception and design of the research. MV: recruitment. MV, GC, FP, and AP: data collection. AP, GC, FP, GG, GPa, and AM: protocol implementation. MV and RP: statistical analysis, creation of the tables, and figures. MV and AM: writing of the manuscript. MV, GPa, AP, RP, GG, FP, GC, GPr, and AM: review of the manuscript.

### Conflict of Interest

The authors declare that the research was conducted in the absence of any commercial or financial relationships that could be construed as a potential conflict of interest.
