# Supplementary figures and images for: Optical Coherence Tomography in a Cohort of Genetically Defined Hereditary Spastic Paraplegia: A Brief Research Report
Source: Front Neurol. 2019 Nov 22;10:1193. doi: 10.3389/fneur.2019.01193 (PMC6884025; doi:10.3389/fneur.2019.01193)

$\Delta$  R-SUP  $\Delta$  R-INF  $\Delta$  R-TEMP  $\Delta$  R-NAS  $\Delta$  L-SUP  $\Delta$  L-INF  $\Delta$  L-TEMP  $\Delta$  L-NAS

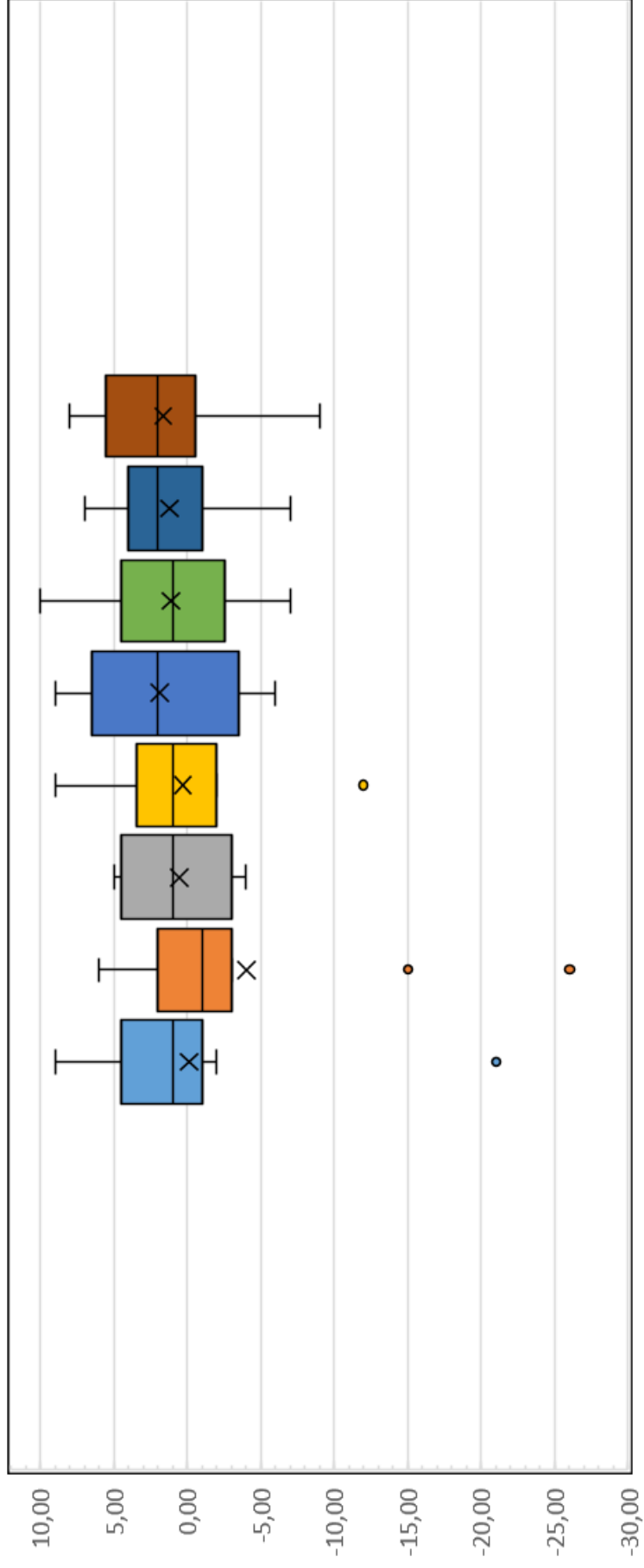

Supplement: Supplementary file 1 [file Data_Sheet_1.pdf]
