# Supplementary material for: Optical Coherence Tomography in a Cohort of Genetically Defined Hereditary Spastic Paraplegia: A Brief Research Report
Source: Front Neurol. 2019 Nov 22;10:1193. doi: 10.3389/fneur.2019.01193 (PMC6884025; doi:10.3389/fneur.2019.01193)
Supplement: Supplementary file 2 [file Data_Sheet_2.pdf]

Supplementary Material

Supplementary Figure 1. Box-and-whiskers of the longitudinal RNFL thickness variation among all the quadrants bilaterally. Legend: RNFL: Retinal Nerve Fibre Layer; R-SUP: right superior; R-INF: right inferior; R-TEMP: right temporal; R-NAS: right nasal; L-SUP: left superior; L-INF: left inferior; L-TEMP: left temporal; L-NAS: left nasal.

Supplementary Table 1. Cross-sectional OCT scan raw data in all the subjects divided according to the genotype. Legend: Green: normal; Yellow: bordeline (< -2 SD); Red: reduced (< -3 SD); R-SUP: right superior; R-INF: right inferior; R-TEMP: right temporal; R-NAS: right nasal; L-SUP: left superior; L-INF: left inferior; L-TEMP: left temporal; L-NAS: left nasal.

	RSUP	RINF	RTEMP	RNAS	LSUP	LINF	LTEMP	LNAS
**SPG3A**	86	97	71	44	87	93	68	36
	114	143	54	82	113	126	69	62
	110	109	79	40	117	114	69	44
	126	121	73	43	132	132	66	53
	105	118	81	50	110	122	76	51
	120	123	80	48	128	112	74	45
**SPG4**	119	135	71	112	134	131	74	100
	124	110	62	76	131	106	59	71
	126	112	68	64	106	86	54	43
	84	100	61	68	76	104	63	62
	118	131	85	72	120	132	76	78
	120	137	70	83	109	143	69	78
	98	110	73	55	110	113	62	59
	80	106	57	61	86	109	54	63
**SPG5**	110	86	67	53	122	78	63	67
	103	110	45	61	111	120	44	71
**SPG7**	92	92	64	46	94	97	56	48
	119	132	53	83	130	123	62	65
**SPG8**	111	116	67	63	110	120	67	58
	104	118	94	68	94	86	69	58
**SPG72**	117	104	74	76	126	118	68	77
	114	134	77	99	112	138	75	109
	84	111	77	68	117	134	67	78

Supplementary Table 2. Correlations of ranked variable values between the clinical and RNFL thickness in all quadrants bilaterally (Spearman rank order correlation). Legend: DD: disease duration; SPRS: Spastic Paraplegia Rating Scale; RNFL: Retinal Nerve Fibre Layer; Correlation is significant at the 0.05 level (2-tailed) as * and at 0.01 level (2-tailed) as **. Significant correlations appear in bold font.

Variables	R-SUP (Rho; Sig)	R-INF (Rho; Sig)	R-TEMP (Rho; Sig)	R-NAS (Rho; Sig)	L-SUP (Rho; Sig)	L-INF (Rho; Sig)	L-TEMP (Rho; Sig)	L-NAS (Rho; Sig)
**Age at OCT**	-0,110; 0,616	-0,121; 0,582	-0,140; 0,524	0,332; 0,122	-0,023; 0,918	-0,067; 0,766	-0,260; 0,231	**0,516*; 0,012**
**Age at Onset**	-0,162; 0,461	-0,373; 0,080	-0,120; 0,585	0,193; 0,378	-0,068; 0,758	-0,187; 0,404	-0,392; 0,064	0,370; 0,082
**DD**	0,143; 0,517	**0,574**; 0,004**	0,171; 0,436	0,158; 0,472	0,015; 0,946	0,293; 0,185	**0,436*; 0,037**	0,010; 0,964
**SPRS**	0,232; 0,288	0,328 0,126	0,196; 0,370	-0,196, 0,370	0,073; 0,740	-0,037; 0,869	0,221; 0,311	-0,349; 0,102
